# Subspecies-specific sequence detection for differentiation of *Mycobacterium abscessus* complex

**DOI:** 10.1038/s41598-020-73607-x

**Published:** 2020-10-02

**Authors:** Alina Minias, Lidia Żukowska, Jakub Lach, Tomasz Jagielski, Dominik Strapagiel, Su-Young Kim, Won-Jung Koh, Heather Adam, Ruth Bittner, Sara Truden, Manca Žolnir-Dovč, Jarosław Dziadek

**Affiliations:** 1 Institute of Medical Biology, Polish Academy of Sciences, ul. Lodowa 106, 93-232 Lodz, Poland; 2 BioMedChem Doctoral School of the University of Lodz, The Institutes of the Polish Academy of Sciences, Lodz, Poland; 3 Biobank Lab, Department of Molecular Biophysics, Faculty of Biology and Environmental Protection, University of Lodz, Lodz, Poland; 4 Department of Medical Microbiology, Institute of Microbiology, Faculty of Biology, University of Warsaw, Warsaw, Poland; 5 Division of Pulmonary and Critical Care Medicine, Department of Medicine, Samsung Medical Center, Sungkyunkwan University School of Medicine, Seoul, South Korea; 6 Diagnostic Services, Shared Health, Winnipeg, MB Canada; 7 National Reference Laboratory for Mycobacteria, University Clinic of Respiratory and Allergic Diseases, Golnik, Slovenia

**Keywords:** Infectious-disease epidemiology, Epidemiology

## Abstract

*Mycobacterium abscessus* complex (MABC) is a taxonomic group of rapidly growing, nontuberculous mycobacteria that are found as etiologic agents of various types of infections. They are considered as emerging human pathogens. MABC consists of 3 subspecies—*M. abscessus* subsp. *bolletti*, *M. abscessus* subsp. *massiliense* and *M. abscessus* subsp. *abscessus*. Here we present a novel method for subspecies differentiation of *M. abscessus* named Subspecies-Specific Sequence Detection (SSSD). This method is based on the presence of signature sequences present within the genomes of each subspecies of MABC. We tested this method against a virtual database of 1505 genome sequences of MABC. Further, we detected signature sequences of MABC in 45 microbiological samples through DNA hybridization. SSSD showed high levels of sensitivity and specificity for differentiation of subspecies of MABC, comparable to those obtained by *rpoB s*equence typing.

## Introduction

*Mycobacterium abscessus* complex (MABC) is a taxonomic group of rapidly growing, nontuberculous mycobacteria. They are emergent human pathogens implicated in a variety of clinical manifestations. *Mycobacterium abscessus* ranks fifth as the most commonly isolated nontuberculous mycobacterial (NTM) species from pulmonary samples worldwide^1^.

Due to the difficulties in clinical management, these infections have earned them the label “clinical nightmare”^2,3^. Pulmonary disease is the most common clinical presentation, especially among cystic fibrosis (CF) patients^4,5^. Wound and skin infections, typically associated with cosmetic surgery and other iatrogenic procedures, have also been reported^6,7^. While the MABC infections are relatively rare, though, with rising incidence, they pose a serious therapeutic challenge^2,8,9^. MABC infections have limited treatment modalities due to both intrinsic and acquired drug resistance^2^. The treatment outcomes are often poor. The cure rate, defined as persistent culture conversion, usually ranges between 30 and 50%^10^.

MABC consists of three subspecies, namely *M. abscessus* subsp. *abscessus*, *M. abscessus* subsp. *bolletti* and *M. abscessus* subsp. *massiliense*^11^. The contribution of each subspecies to the development and the outcome of the disease is unclear, yet certain differences have been observed. First, based on data available from the United States and Europe, infections with *M. abscessus* subsp. *bolletii* are much rarer when compared with two other subspecies^5,12,13^, suggesting their different levels of adaptation for pathogenic lifestyle. Second, infections due to *M. abscessus* subsp. *massiliense* respond more favorably to antimicrobial therapy^14,15^. Both, *M. abscessus* subsp*. abscessus* and *M. abscessus* subsp. *bollettii,* possess a functional erythromycin ribosome methyltransferase gene *erm(41)* responsible for macrolide resistance^14,16^. In *M. abscessus* subsp. *massiliense* the *erm(41)* gene is truncated and thus non-functional.

In guidelines issued in 2007, the American Thoracic Society recommends the identification of infecting non- tuberculous bacterial species^17^. The British Thoracic Society recommends subspecies identification of MABC in guidelines issued in 2017^18^. Several sequence-based methods allow the identification of the three MABC subspecies. Typically, these methods use sequencing of several housekeeping genes, including *hsp65*, *rpoB, secA*, either individually^19–22^, or in a combined manner^23^. Moreover, there are several PCR-based methods, i.e., repetitive sequence-based PCR (rep-PCR)^22^, deletion-mapping PCR^24^, and peptide nucleic acid multi-probe-real-time PCR^25^. Most commonly, the detection of deletion is used to differentiate *M. abscessus* subsp. *massiliense* from *M. abscessus* subsp. *bolletii* and *M. abscessus* subsp. *abscessus* by sequencing of the *erm(41)* gene, as it also serves for clarithromycin resistance prediction.

Here we present a novel method for subspecies differentiation of *M. abscessus* named SubSpecies-Specific Sequence Detection (SSSD). This method is based on the presence of signature sequences within the genomes of all MABC subspecies. The method was validated against a virtual database of 1505 MABC genome sequences from across the world, and it was highly effective for subspecies-level discrimination. Furthermore, the method showed to be valid for discrimination of subspecies of MABC in laboratory conditions through DNA hybridization. Its differentiation accuracy in terms of sensitivity and specificity was comparable to that obtained with *rpoB* sequencing. DNA sequencing, as a technique, is rarely available for diagnostic laboratories settled outside the most developed countries of Western Europe and the United States. SSSDs, as a method, forms a solid base for further development with various molecular biology techniques and fills the gap for less developed countries or laboratories with lower resources. The principal of SSSD is the detection of specific coding sequences. These sequences, or products of these genes, can be detected by several techniques, including the simplest PCR, DNA hybridization, or immunodetection. They may also be detected by more sophisticated methods like MALDI-TOF or mass spectrometry, and DNA sequencing. We expect that SSSD will facilitate the differentiation of subspecies of *M. abscessus* in laboratories where specialized equipment is not readily available.

## Results

### Differentiation of genome sequences included in the virtual database

A total of 1505 genomic sequences of *M. abscessus* included in our virtual database, were differentiated by MLST typing coupled with gANI differentiation and SSSD, *rpoB* sequence typing and *erm(41)* sequence typing.

First, we performed virtual MLST typing on all MABC genomes. We used PasteurMLST database of Institute Pasteur to identify alleles and sequence types (STs). We identified 181 distinct MLST patterns, 50 of which could be assigned to already known STs. The remaining 131 patterns had previously been unreported. We named new patterns of MLST with the alphabetic scheme. The three most prevalent STs within our database were ST1, representing *M. abscessus* subsp. *abscessus* (18.5%, n = 279), ST23, representing *M. abscessus* subsp. *massiliense* (16.6%, n = 250), and ST26, representing *M. abscessus* subsp*. abscessus* (10.2%, n = 154).

**Figure 1:**
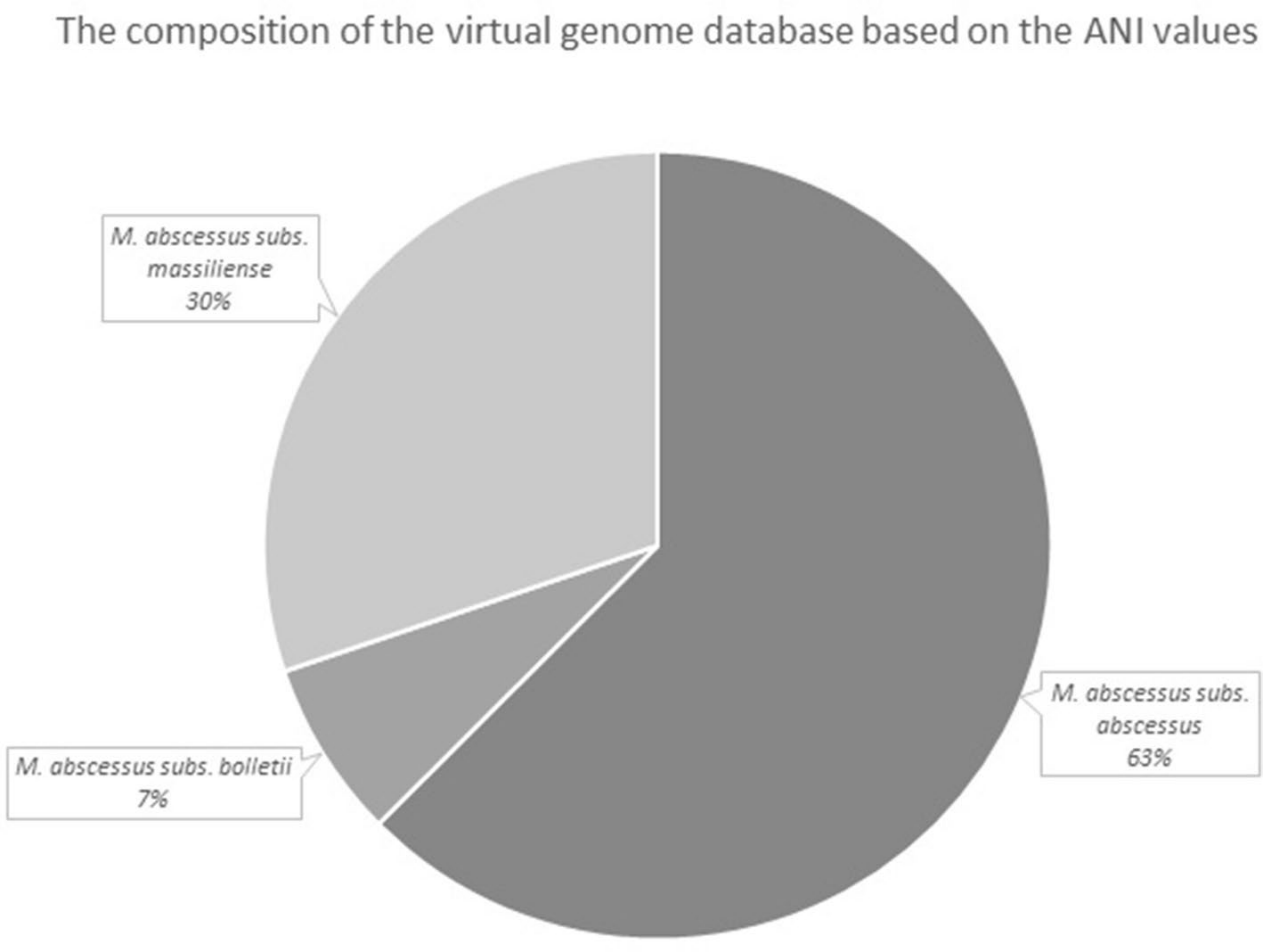
The distribution of strains of *M. abscessus* complex strains included in this study belonging to individual subspecies. The subspecies identification was obtained through the MLST typing, followed by subspecies identification by gANI.

We differentiated a total of 181 M*. abscessus* strains showing unique MLST patterns into individual subspecies based on gANI values. Our reference strains were NC_010397 (ATCC 19977) for *M. abscessus* subsp. *abscessus*, NC_018150 for *M. abscessus* subsp. *massiliense* and NZ_CP014950 for *M. abscessus* subsp. *bolletti*. The strains included in the database were clustered into three separate groups (Figs. S6, S7), corresponding to individual subspecies. Similar to previous reports^11^, the intrasubspecies ANI values exceeded 98%, while intersubspecies ANI values ranged from 96 to 98% (Table S2). The ANI values for the most closely related species of mycobacteria*, Mycobacterium chelonae*, *Mycobacterium porcinum*, *Mycobacterium farcinogenes*, *Mycobacterium fortuitum*, and *Mycobacterium immunogenum* ranged between 74 and 88%. Based on combined MLST and ANI score differentiation, we identified 63% (n = 941) strains in the database as *M. abscessus*. subsp. *abscessus*, 30% (n = 454) as *M. abscessus* subsp. *massiliense* and 7% (n = 110) as *M. abscessus. abscessus* subsp. *bolletii* (Fig. 1). We used ANI identification as the gold standard, which allowed us to estimate the sensitivity and specificity of the SSSDs and other methods used for subspecies identification.

**Figure 2:**
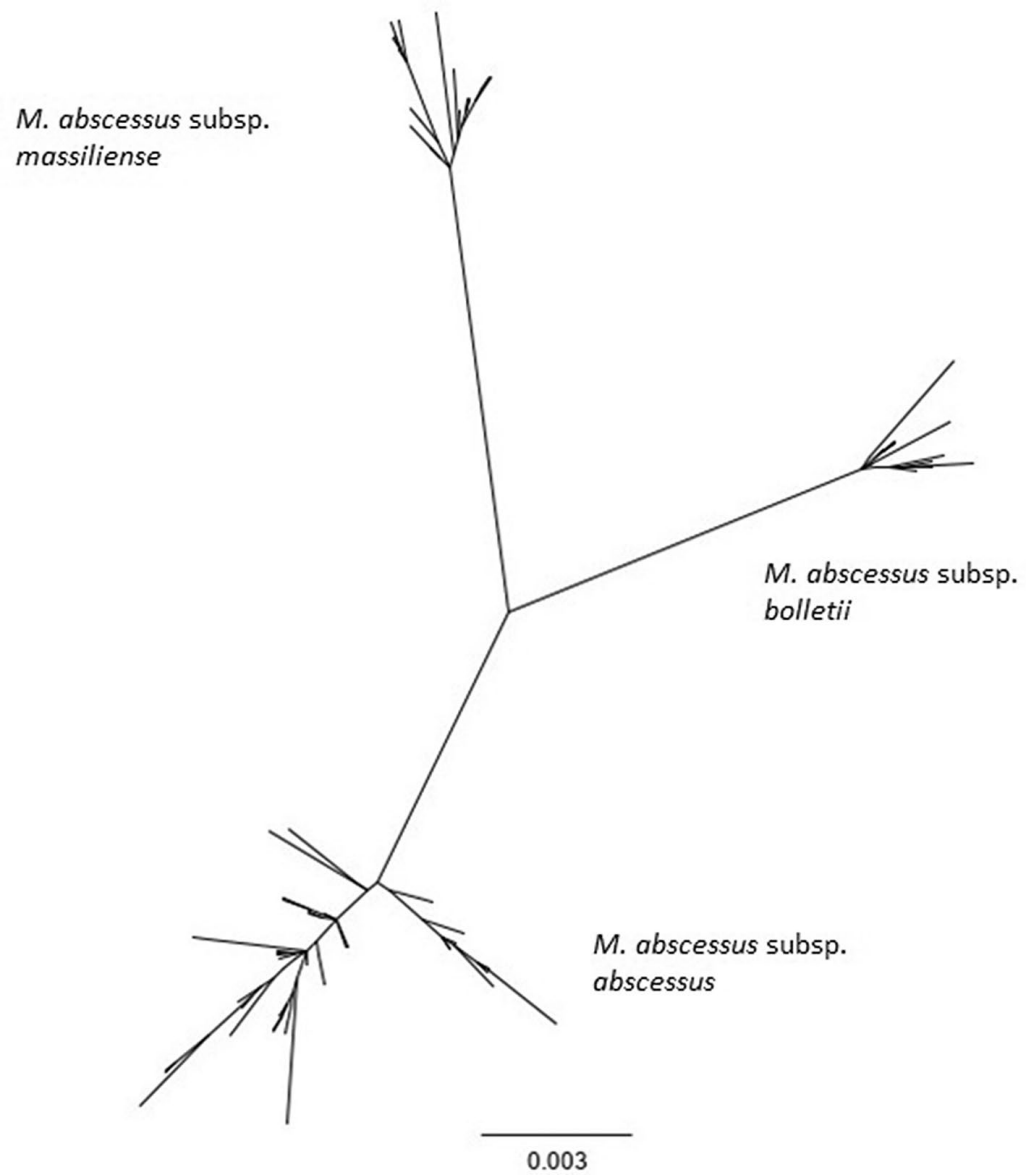
Phylogenetic RAxML tree built on *rpoB* sequences found among the population of 1505 strains of MABC using Geneious R11 software. Each tip of the tree represents a unique sequence of the *rpoB* gene. The phylogenetic branches of the tree represent distinct subspecies of MABC- *M. abscessus* subsp. *abscessus*, *M. abscessus* subsp. *bolletii* and *M. abscessus* subsp. *massiliense.*

Upon full-length *rpoB* sequence typing of genomes included in the virtual MABC genome database, we observed 97 distinct *rpoB* alleles. There were 213 variable sites within this gene, with a total of 220 mutations. The average number of nucleotide differences was 38.118. All *rpoB* gene sequevars, including those of three reference strains of MABC subspecies, grouped into three branches on the phylogenetic tree (Fig. 2). We found 939 strains of *M. abscessus* subsp. *abscessus*, 456 strains of *M. abscessus* subsp. *massiliense*, and 110 strains of *M. abscessus* subsp. *bolletii*. Both sensitivity and specificity of *rpoB* typing for differentiation of MABC subspecies were high, exceeding 98% (Table 1). Overall, *rpoB* typing enabled accurate identification of 99.1% (n = 1491) of MABC strains.

**Table 1:** Sensitivity and specificity values of differentiation methods tested within this study.

	Sensitivity		Specificity	
	Value (%)	95% CI	Value (%)	95% CI
***rpoB typing***				
*M. abscessus* subs. *abscessus*	99.2	98.3–99.6%	98.9	97.7–99.6%
*M. abscessus* subs. *bolletii*	100	96.7–100%	100	99.7–100%
*M. abscessus* subs. *massiliense*	98.7	97.2–99.5%	99.2	98.5–99.7%
***erm41 typing***				
*M. abscessus* subs. *abscessus*	96.9	95.6–97.9%	97.6	95.7–98.8%
*M. abscessus* subs. *bolletii*	100	96.7–100%	100	71.5–100%
*M. abscessus* subs. *massiliense*	97.6	95.7–98.8%	97.2	96.1–98.1%
**SSSD**				
*M. abscessus* subs. *abscessus*	96.7	95.4–97.7%	100	99.3–100%
*M. abscessus* subs. *bolletii*	87.3	79.6–92.9%	100	99.7–100%
*M. abscessus* subs. *massiliense*	100	99.2–100%	97.2	96–98.1%

As with *rpoB* typing, sequencing using the *erm(41*) gene also showed high levels of sensitivity and specificity (> 97%), but this method did not differentiate *M. abscessus* subsp. *abscessus* from *M. abscessus* subsp. *bolletii* (Table 1). We found 29 false-positive results for *M. abscessus* subsp*. massiliense* and 11 false-negative strains. These values reversed for other MABC subspecies, hence 29 false-negative and 11 false-positive cases of identification of *M. abscessus* subsp. *abscessus/bolletii*. Overall, *erm(41)* typing identified properly 98.1% (n = 1476) of MABC strains at the subspecies level.

For SSSD differentiation, our custom virtual database of MABC genome sequences was BLAST-searched to identify sequences specific for each of the MABC subspecies. We typed 1489 (98.9%) out of 1505 genomes at the subspecies level. We obtained ambiguous results for 16 genomes. These genomes represented four MLST patterns. For two MLST patterns, ST109 type (n = 1), and new MLST pattern (DF) (n = 13), the ambiguities resulted from simultaneous detection of bol-s-s and mas1-s-s or mas2-s-s. gANI analysis identified these strains as *M. abscessus* subsp. *bolletii*. One strain, new MLST pattern (BD), was recognized by mas1-s-s and abs-s-s, and one strain, new MLST pattern (CL), was recognized by mas2-s-s and abs-s-s. gANI identified both these strains as *M. abscessus* subsp. *abscessus*.

Excluding strains with ambiguous results, a sequence-specific for *M. abscessus* subsp. *abscessus* (abs-s-s) was found in 910 (60.5%) genomes, whereas sequence specific for *M. abscessus* subsp. *bolletii* (bol-s-s)—in 96 genomes. Sequences mas1-s-s and mas2-s-s were detected in 431 and 467 genomes, respectively. We concurrently detected both sequences specific for *M. abscessus* subsp. *massiliense* in 399 strains. Either of the two sequences were found in 483 strains of *M. abscessus* subsp. *massiliense*.

Only when using SSSD for *M. abscessus* subsp. *massiliense* some false-positive results appeared. We detected a mas2 sequence in 29 genomes, found to represent *M. abscessus* subsp. *abscessus* upon gANI analysis. These genomes were assigned to three MLST types, i.e.: MLST ST37 (n = 27), MLST ST60 (n = 1) and new MLST pattern (CJ) (n = 1). Of the total 31 genomes that harbored ST37, 27 were *M. abscessus* subsp*. abscessus*, while only four—*M. abscessus* subsp. *massiliense*. Two genomes represented ST60, and each was assigned to different subspecies by gANI. *erm(41)* typing assigned *M. abscessus* subsp. *massiliense* to all of the genomes that SSSD, but not gANI, assigned as *M. abscessus* subsp. *massiliense*. Six of those genomes were assigned as *M. abscessus* subsp. *massiliense* based on *rpoB* typing while the remaining 23 were recognized as *M. abscessus* subsp. *abscessus*.

Overall, SSSD produced specificities and sensitivities of 100% and 96.7% for *M. abscessus* subsp. *abscessus*, 100% and 87.3% for *M. abscessus* subsp. *bolletii*, and 97.2% and 100% for *M. abscessus* subsp. *massiliense*, respectively (Table 1). With SSSD, we obtained an accurate, subspecies-level identification for 97% (1460) of the MABC genomes.

### Validation of SSSD on clinical isolates

**Figure 3:**
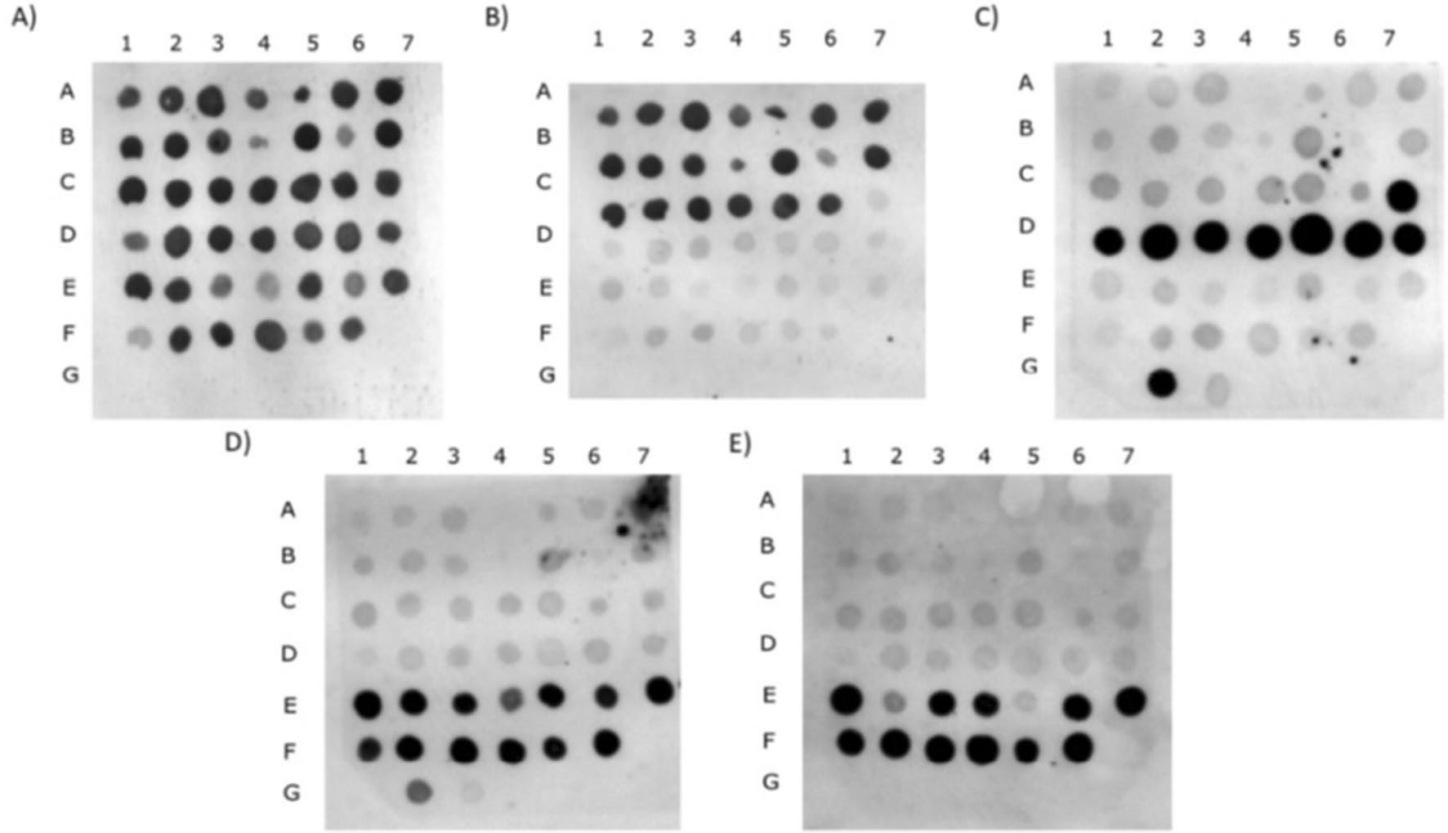
Dot blot analysis of chromosomal DNA hybridization with MABC. The isolated DNA was placed on Hybond-N + membrane in the following order: A1–C6 *M. abscessus* subsp. *abscessus* strains, C7–D7 *M. abscessus* subsp. *bolletii* strains, E1–F6 *M. abscessus* subsp. *massiliense* strains, G2–G3 *M. chelonae,* G4 *M. fortuitum,* G5 *M. porcinum*. The blots were hybridized with labelled probes targeting (**A**) MABC-s-s, (**B**) abs-s-s, (**C**) bol-s-s, (**D**) mas1-s-s, (**E**) mas2-s-s.

The SSSD method was validated using 45 strains of MABC collected in three countries—Canada, South Korea, and Slovenia and three closely related species of *M. chelonae* (two strains)*, M. fortuitum* (one strain)*,* and *M. porcinum* (one strain). The strains were typed using methods described in the Materials and Methods section of this manuscript. The probes targeting MABC-s-s and abs-s-s were 100% sensitive and specific (Fig. 3A,B, respectively ). The probes bol-s-s and mas1-s-s detected specific sequences in all respective strains and one strain of *M. chelonae* (Fig. 3C,D, respectively). Probe hybridizing to mas2-s-s detected 11 out of 13 strains (84%) of *M. abscessus* subsp*. massiliense* (Fig. 3E). Overall, the method of SSSD correctly typed species and subspecies of all clinical isolates included in this study (Table 2).

**Table 2:** Sensitivity and specificity values of SSSD performed on 41 clinical isolates of *M. abscessus* complex.

	Sensitivity		Specificity	
	Value (%)	95% CI	Value (%)	95% CI
**SSSD**				
*M. abscessus* subs. *abscessus*	100.00	83.16–100.00%	100.00	83.89–100.00%
*M. abscessus* subs. *bolletii*	100.00	63.06–100.00%	100.00	89.42–100.00%
M. *abscessus* subs. *massiliense*	100.00	86.77–100.00%	100.00	93.62–100.00%

## Discussion

The population of strains included within the virtual database reflects the population of infecting strains, with the highest prevalence of strains of M. *abscessus* subsp. *abscessus*, followed by *M. abscessus* subsp. *massiliense* and *M. abscessus* subsp. *bolletii*. Among 1505 genome sequences, we identified 131 MLST patterns that were previously unreported, which shows that there is still a significant level of variability to be discovered within the genus of *M. abscessus*.

The SSSD developed in this study reached satisfactory levels of sensitivity and specificity upon in silico, genome-based analysis, and validation procedure on DNA from 45 clinical isolates. When we validated SSSD on clinical strains, we found that the probes detecting bol-s-s and mas1-s-s detected specific sequences in all respective strains and one strain of *M. chelonae*. We suspect a possibility of horizontal gene transfer between *M. abscessus* and *M. chelonae*. The sequences that we detected in this study are present in only one out of three subspecies of *M. abscessus* complex. We suspect that these sequences were not present in the ancestral strain of *M. abscessus* complex, and ancestral strains of each subspecies might have acquired them from other bacteria during the process of phylogenetic differentiation. Due to possible cross-reactivity of SSSDs in other species, we recommend the use of *M. abscessus* specific sequence to identify species of the bacteria, together with subspecies specific sequences. Relying only on the detection of subspecies specific sequences only may result in erroneous identification.

We found that the results obtained by SSSD were not utterly concordant with the results obtained with gANI. We made the same observation for other methods of strain differentiation, *rpoB* sequencing, and *erm(41)* typing. We suspect that it is a result of the horizontal gene transfer between bacteria within the complex. This phenomenon was suggested as an important factor contributing to the evolution of *M. abscessus*^26,27^. *M. abscessus* is considered a pathogen with open, non-conservative pangenome, which means it should be monitored closely for the acquisition of pathogenic traits^28^. Tan et al. found that recombination MABC more than mutations and natural selection. After a comparison of 243 genomes of *M. abscessus,* they found that the recombined sequences in the three subspecies have come from different intra-species and inter-species origins^27^. The observations of Tan et al. reflected in our study. There are reports that recombination occurs more frequently in *M. abscessus* subsp. *massiliense* than in the other two subspecies^27^, which can explain why two subspecies specific sequences are necessary to discriminate M. abscessus subsp efficiently. *massiliense* from other members of the complex. Further, for all typing methods, sequences that are, in general, specific for one subspecies, have been identified in bacteria, ultimately belonging to distinct subspecies.

The genetic distinction of the subspecies within the complex, suggests the existence of reproductive isolation barriers between bacteria. The extent of these barriers, their mechanism of action, both within and outside the complex, remain to be understood. Importantly, during this research, we observed cross-hybridization of the sequences that detected genes of subspecies of MABC (bol-s-s and mas1-s-s) in a clinical strain of *M. chelonae* during DNA hybridization. Perhaps, the reproductive barriers are permeable not only between the subspecies of MABC but also between closely related species of mycobacteria. The existence of horizontal gene transfer is a potential limitation of the accuracy of the method that we present.

One of the most significant advantages of SSSD is that the method relies on the identification of a stretch of a particular coding DNA sequence. Therefore, this method is adaptable to a simple, time- and cost-efficient method, which can be an alternative for sequencing-based methods of differentiation in laboratory conditions. In this study, we used DNA hybridization to show that SSSD is efficient in discriminating subspecies of MABC in laboratory conditions. The protocol involved growing bacteria on agar plates, DNA isolation, and DNA hybridization. The entire experiment took approximately a week. However, this method is amenable to more time- and cost-efficient protocols with other molecular biology techniques, for example, microarrays^29^ or line probe assays based on reverse hybridization^30^. Subspecies specific sequences can easily be detected using the simplest PCR. Importantly, as showed by the results of this study, the method of SSSD can be exploited for quick and efficient analysis of data obtained by whole-genome sequencing. The bioinformatic methods based on the comparison of entire genomes, like gANI, still require large amounts of computer power, which is neither practical nor accessible for many laboratories. In turn, the BLAST search of the sequence of interest takes seconds. Finally, the use of coding sequences for differentiation allows the possibility to identify *M. abscessus* and its subspecies in antigen-based cassette tests. The principal of the method fills the gap for *M. abscessus* identification to the subspecies level, where DNA sequencing is not available.

## Materials and methods

### Construction of virtual database

Genome sequences of 1505 M*. abscessus* strains were downloaded from Genome Database at the NCBI. Information on the geographic origin of the strains was retrieved from the Genome Database (Table S1). The strains included in the database were isolated in 14 countries from across the globe (Fig. S1, Table S1). All genome sequences consisted of less than 145 contigs in order to ensure good quality of extracted sequences. The cut-off for the number of contigs, for each strain, was linked to the N50 value, typically used to describe the quality of draft assembly. The N50 value is defined as the shortest sequence length at 50% of the genome. The lowest probable N50 value in our dataset was 35 kbp, with an average N50 of 384.13 ± 18.06 kbp.

### Virtual database strain differentiation

The sequences were processed and analyzed with Geneious R11 (Biomatters, Auckland, New Zealand)^31^. We compiled genomic sequences of *M. abscessus* strains into a custom database. We searched the database with the BLAST tool implemented in Geneious R11 (Megablast, max e-value 1e−4) for the presence of the *rpoB*, *erm(41)* gene sequences and another seven gene sequences (*argH*, *cya*, *glpK*, *gnd*, *murC*, *pta* , and *purH*.) used in the MLST typing. Reference sequences for BLAST search were those of strains NC_010397 (reference strain *M. abscessus* ATCC 19977) for *M. abscessus* subsp. *abscessus*, NZ_CP014950 for *M. abscessus* subsp. *bolletii,* and NC_018150 for *M. abscessus* subsp. *massiliense*. Sequences found through BLAST search were aligned with Geneious R11. The strains were differentiated to the subspecies level based on *rpoB* and *erm(41)* sequence typing and MLST typing, as previously described^32^. For *rpoB* sequences, a phylogenetic tree was built with RAxML implemented within Geneious R11. Sequence variability was analyzed with DnaSP^33^. For *erm(41)*, strains were assigned to individual subspecies upon analysis of length of the sequences found during BLAST search, as *erm(41)* sequence typing differentiates *M. abscessus* subs. *massiliense* from other members of the complex based on the absence of the C-terminal end of the gene. For SSSD, strains were categorized into individual subspecies, based on the presence of particular sequences of a given strain (Table 3). These sequences were identified on a hit list after the BLAST search of the custom virtual *M. abscessus* database. The reference sequence used to identify *M. abscessus* subsp. *abscessus* (*M. abscessus* subsp. *abscessus*-specific sequence, abs-s-s) was a gene sequence encoding hypothetical protein MAB_3505c. For identification of *M. abscessus* subsp. *bolletii* we used sequence of *aac1* gene encoding ADP/ATP carrier protein (bol-s-s), located between homologs of MAB_1240 and MAB_1241c. For identification of *M. abscessus* subsp. *massiliense* we used two sequences. The first sequence (mas1-s-s) was annotated as a steroid dehydrogenase and was located between homologs of MAB_1115 and MAB_1116. The second sequence (mas2-s-s) was annotated as *tetR* gene and located within homolog of MAB_2150c.

**Table 3:** Genomic location of probe-binding sites.

Probe	Gene	Size of the gene (bp)	Location in reference to NC_010397 *M. abscessus* subsp. *abscessus*	Locus tag	Predicted gene function
abs-s-s	Hypothetical	399	MAB_3505c	MAB_3505c	Unknown
bol-s-s	*aac*1	2358	Between homologs of MAB_1240 and MAB_1241c	A3N95_RS05745	ADP/ATP carrier protein
mas1-s-s	DUF1295 domain-containing protein	762	Between homologs of MAB_1115 and MAB_1116	MYCMA_RS17980	Steroid dehydrogenase
mas2-s-s	*tet*R	600	Within homolog of MAB_2150c	MYCMA_RS13635	Transcriptional regulator

MLST patterns were assigned sequence type (ST) numbers from publically available database PubMLST.org^34^ (Table S1). In order to designate the subspecies for each MLST pattern, strains showing distinct MLST patterns were chosen for subspecies delineation by genome average nucleotide identity estimation (gANI)^11,35^. gANI is a similarity index between a given pair of genomes. Briefly, 181 genomes of *M. abscessus* showing distinct MLST patterns were annotated with DFAST^36^. Predicted coding sequences were used for calculating pairwise gANI with ANIcalculator^37^ (Fig. S2, Table S2). Data processing and visualization were performed with Python 3 scripts using pandas and seaborn libraries. Additional ANI analysis was performed for 47 genomes of MABC, where subspecies delineation results were not concordant for all methods (Fig. S3, Table S3). In the case of discrepancies, the delineation of species by ANI was considered as correct.

### Bacterial strains

All strains were isolated from pulmonary patients from hospitals in South Korea, Canada, and Slovenia (Table S4). Each strain was assigned to adequate subspecies by either *hsp65*, *rpoB* gene sequencing, 16S rRNA gene, and internal transcribed spacer sequence, or GenoType NTM-DR Hain's test. Among 45 strains used in this study, 20 were identified as *Mycobacterium abscessus* subsp. *abscessus*, 13 were identified as *Mycobacterium abscessus* subsp. *massiliense*, eight were identified as *Mycobacterium abscessus* subsp. *bolletii*. Four were phylogenetically closely related strains of *Mycobacterium chelonae*, *Mycobacterium fortuitum,* and *Mycobacterium porcinum*.

### DNA hybridization

Bacteria were grown on nutrient broth agar plates for 3–5 days at 37 °C. Cells were mechanically disrupted by bead beating, and genomic DNA was isolated with DNAzol (Invitrogen, Carlsbad, USA). Next, 3 μg of each DNA sample, denatured by heating, was loaded onto Hybond-N + membrane (GE Healthcare Life Sciences, Amersham, UK) and crosslinked with UV Stratalinker 1800 (Stratagene, San Diego, USA). DNA probes for DNA hybridization were amplified with *Taq* polymerase (Sigma-Aldrich, Missouri, USA) (Table 4). PCR products were purified using the QIAEXII Gel Extraction Kit (Qiagen, Hilden, Germany) (Figs. S4–S7). The specific sequence for the detection of MABC was described previously^38^. For the detection of MABC, we used a probe for gene encoding uncharacterized protein MAB_0974. For SSSD of subspecies of MABC, we used probes hybridizing to abs-s-s encoding hypothetical protein MAB_3505c, bol-s-s, located between homologs of MAB_1240 and MAB_1241c, mas1-s-s annotated as a steroid dehydrogenase and located between homologs of MAB_1115 and MAB_1116, and mas2-s-s annotated as *tetR* gene and located within homolog of MAB_2150c. Probes were amplified with DNA of strains *M. abscessus* subsp. *abscessus* A10, *M. abscessus* subsp. *bolletii* B5, and *M. abscessus* subsp. *massiliense* M9 and included in this study. Probes were labeled with AlkPhos Direct Labelling and Detection System (GE Healthcare Life Sciences, Amersham, UK). Various temperatures and salt concentrations in buffers were used during DNA hybridization in order to achieve high stringency of the blot. For detection of MABC and *M. abscessus* subsp. *abscessus*, membranes with sample DNA were hybridized with labeled probes at 85 °C overnight in hybridization buffer (AlkPhos Direct Labelling and Detection System protocol, GE Healthcare Life Sciences, Amersham, UK). Next, membranes were washed in primary and secondary wash solutions according to the manufacturer’s instructions. Afterward, membranes were incubated with detection reagent (CDP-Star, GE Healthcare Life Sciences, Amersham, UK) and gently agitated for 5 min. The membranes were then placed in the cassette with a radiography film (CL-XPosure Film, ThermoFisherScientific, Massachusetts, USA) for one hour and developed with Medical X-ray Processor (Kodak, Rochester, USA). For detection of *M. abscessus* subsp. *bolletii* and M*. abscessus* subsp. *massiliense* (probes mas1 and mas2) hybridization was performed at 75 °C. Also, the NaCl concentration in hybridization buffer, primary, and secondary washing solution was lowered to 50 mM and 15 mM, respectively.

**Table 4:** Primers used in this study.

Target	Forward primer	Reverse primer
MABC-s-s	5′ TATGCACGTAGGTATT	5′ ATCAAGTCGGTACTACT
abs-s-s	5′ CTGATATCACCTTGG	5′ ATGTGATCGAAATGTA
bol-s-s	5′ ACGAATACCATATCG	5′ CATTGAAGACGTAATC
mas1-s-s	5′ CTTACAGAGCTGTATCA	5′ TACAGATAGTGGCACT
mas2-s-s	5′ ACTGTTTGAATTGATG	5′ AATAATGTAGGCATGA

## Supplementary information

Supplementary Figures

Supplementary Tables

## Data Availability

All data generated or analysed during this study are included in this published article (and its Supplementary Information files).
