# Supplementary material for: Subspecies-specific sequence detection for differentiation of Mycobacterium abscessus complex
Source: Sci Rep. 2020 Oct 2;10:16415. doi: 10.1038/s41598-020-73607-x (PMC7532137; doi:10.1038/s41598-020-73607-x)
Supplement: Supplementary file 1 — Supplementary Figures [file 41598_2020_73607_MOESM1_ESM.pdf]

Subspecies-specific sequence detection for differentiation of *Mycobacterium abscessus* complex

Alina Minias [1*], Lidia Żukowska [2], Jakub Lach [3], Tomasz Jagielski [4], Dominik Strapagiel [3], Su-Young Kim [5], Won-Jung Koh [# 5], Heather Adam [6], Ruth Bittner [6], Sara Truden [7], Manca Žolnir-Dovč [7], Jarosław Dziadek [1]

[1] Institute of Medical Biology, Polish Academy of Sciences, Lodz, Poland

[2] BioMedChem Doctoral School of the University of Lodz and the Institutes of the Polish Academy of Sciences in Lodz, Lodz, Poland

[3] Biobank Lab, Department of Molecular Biophysics, Faculty of Biology and Environmental Protection, University of Lodz, Lodz, Poland

[4] Department of Medical Microbiology, Institute of Microbiology, Faculty of Biology, University of Warsaw, Warsaw, Poland

[5] Division of Pulmonary and Critical Care Medicine, Department of Medicine, Samsung Medical Center, Sungkyunkwan University School of Medicine, Seoul, South Korea

[6] Diagnostic Services, Shared Health, Manitoba, Canada

[7] National Reference Laboratory for Mycobacteria, University Clinic of Respiratory and Allergic Diseases, Golnik, Slovenia

[#] deceased

*Corresponding author: Alina Minias; ul. Lodowa 106, 93-232 Lodz, Poland; tel: +48 42 272 36 06, fax: +48 42 27 23 630, email: aminias@cbm.pan.pl

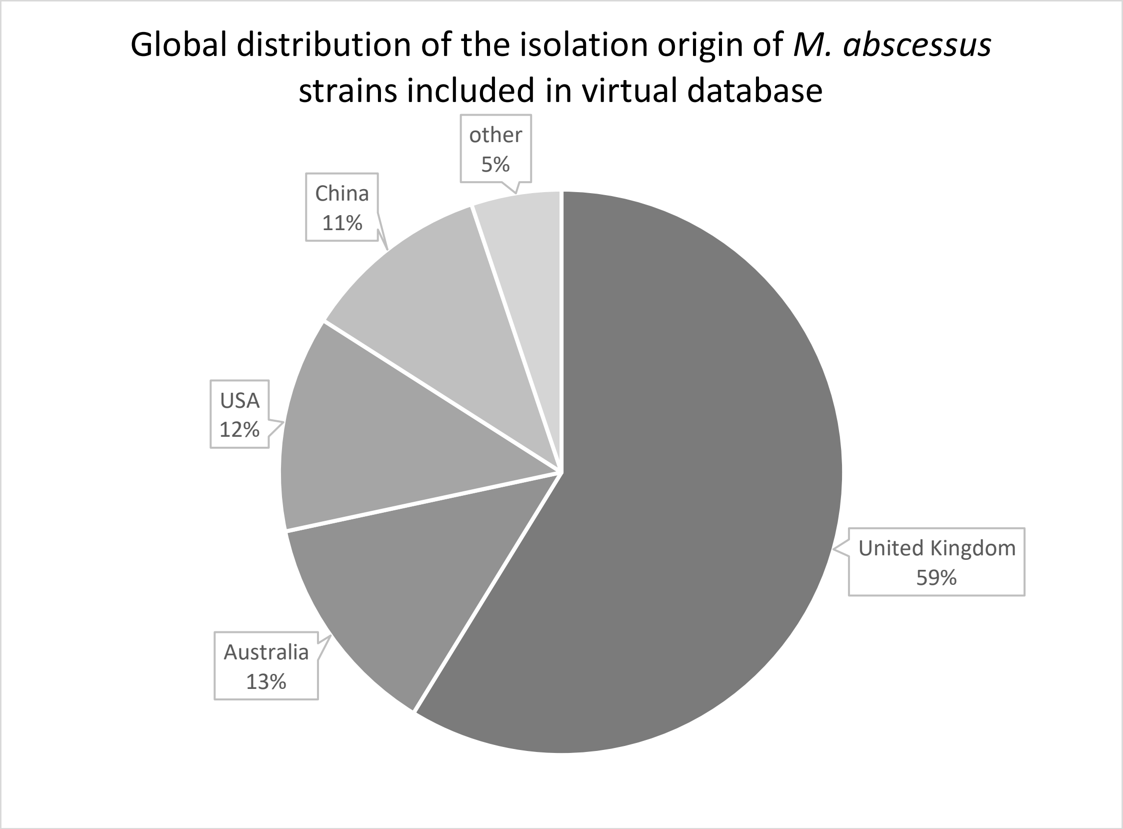

Fig. S1. The geographic origin of the *M. abscessus* strains included in virtual database. The majority of strains included in the database were isolated in United Kingdom. Four continents-Europe, Australia, North America and Asia were represented in the database by at least ten percent of the strains each.

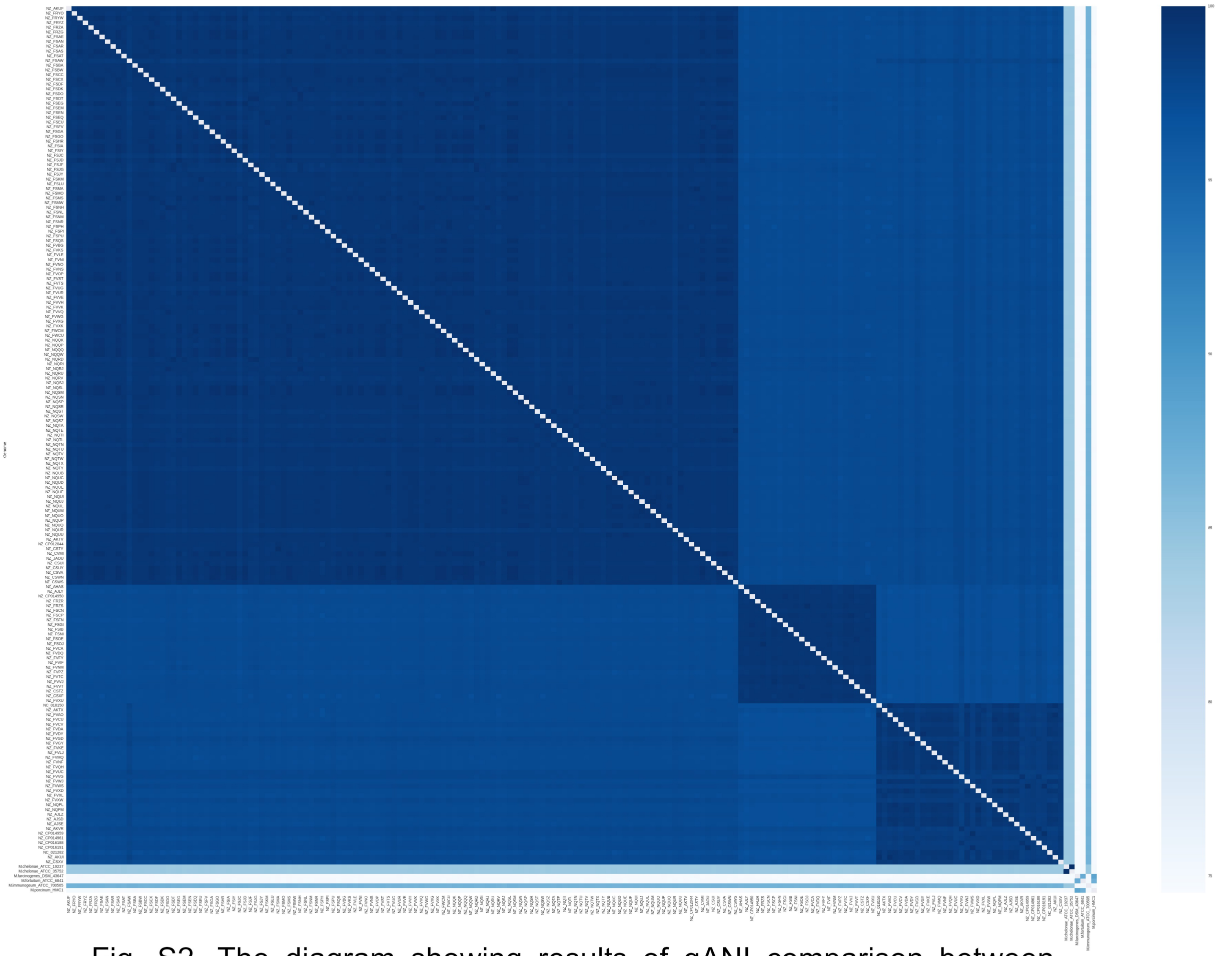

Fig. S2. The diagram showing results of gANI comparison between individual strains of *M. abscessus* complex strains for which the results of differentiation by different methods were inconsistent. The dataset included reference strains for each subspecies- *NC_018150* representing *M. abscessus* subsp. *massiliense, NZ_CP014950* representing *M. abscessus* subsp. *bolletii* and *NC_010397* representing *M. abscessus* subsp. *abscessus.* The blue band on the right of the chart shows the level of similarity between the strains, where dark color represents high similarity of the genomes and light color represents less similar genome sequences.

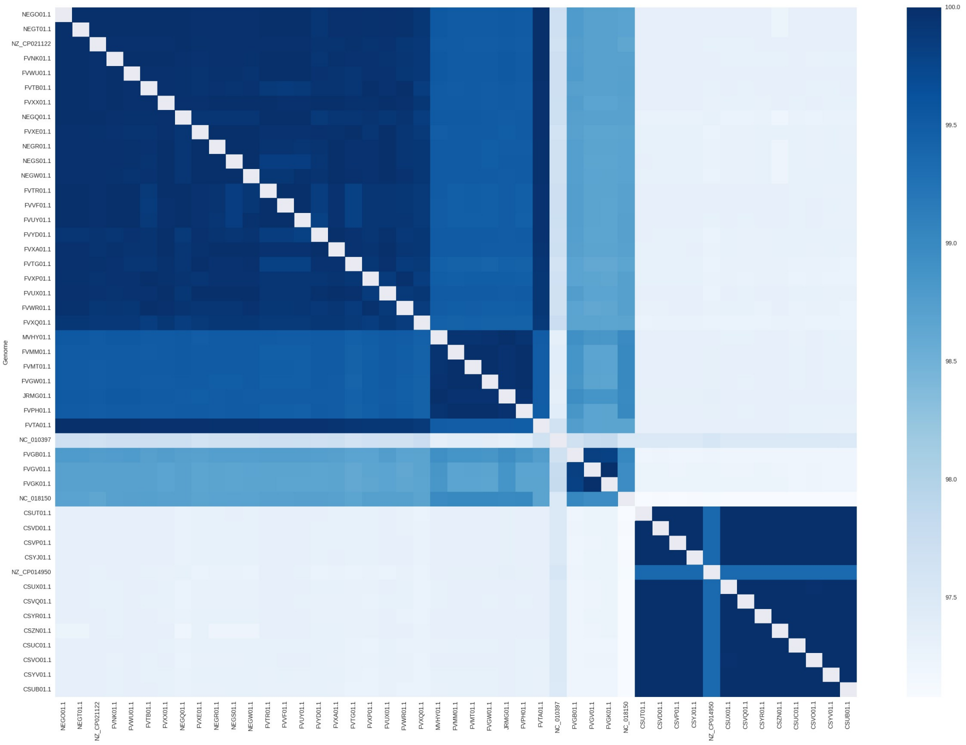

Fig. S3. The diagram showing results of gANI comparison between individual strains of *M. abscessus* complex strains for which the results of differentiation by different methods were inconsistent. The dataset included reference strains for each subspecies- *NC_018150* representing *M. abscessus* subsp. *massiliense, NZ_CP014950* representing *M. abscessus* subsp. *bolletii* and *NC_010397* representing *M. abscessus* subsp. *abscessus.* The blue band on the right of the chart shows the level of similarity between the strains, where dark color represents high similarity of the genomes and light color represents less similar genome sequences.

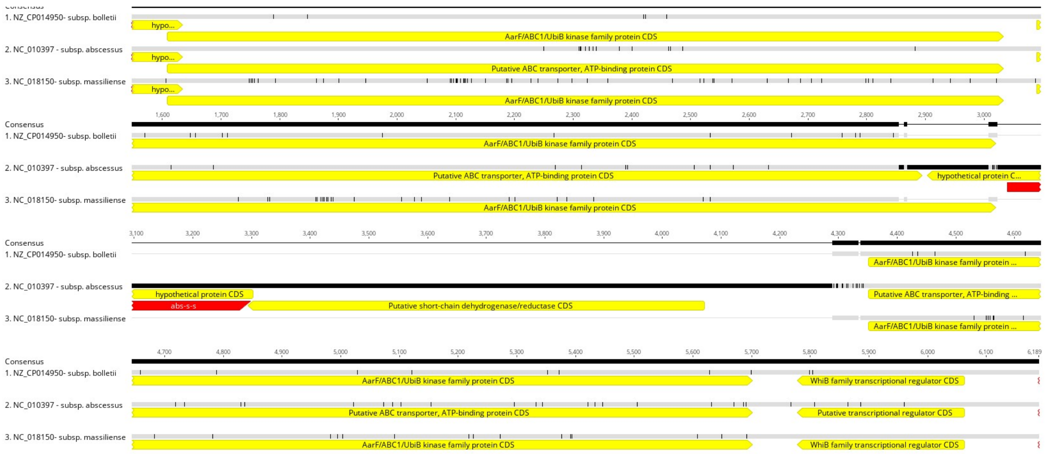

Fig. S4. The alignment between genomic regions surrounding (abs-s-s) sequence in three reference genomes: NC_018150 representing *M. abscessus* subsp. massiliense, NZ_CP014950 representing *M. abscessus* subsp. *bolletii* and NC_010397 representing *M. abscessus* subsp. *abscessus*. Disagreements in the aligned sequences are marked with colored lines. The extent of the genes is marked in yellow. The probe used to detect the sequence in clinical validation of SSSD is marked in red.

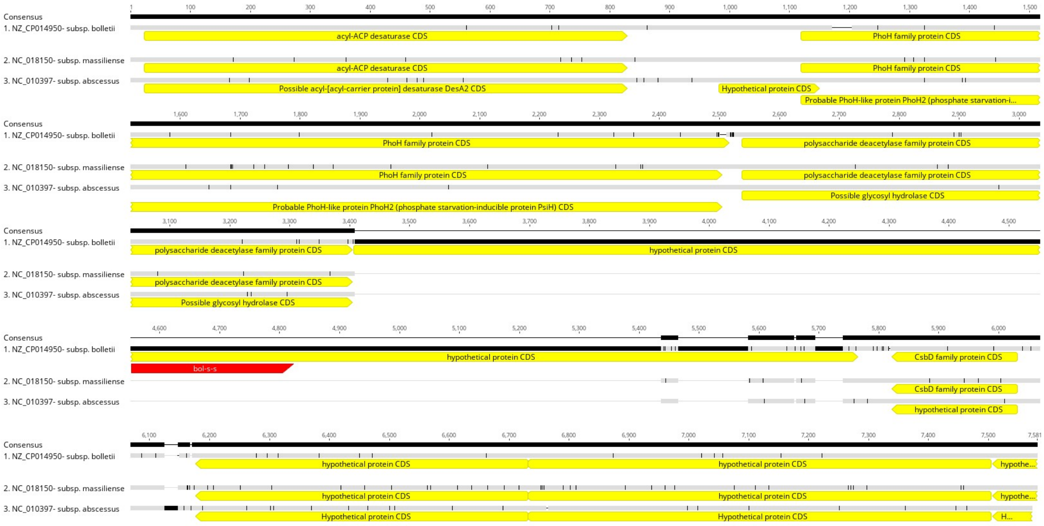

Fig. S5. The alignment between genomic regions surrounding (bol-s-s) sequence in three reference genomes: NC_018150 representing *M. abscessus* subsp. *massiliense*, NZ_CP014950 representing *M. abscessus* subsp. *bolletii* and NC_010397 representing *M. abscessus* subsp. *abscessus*. Disagreements in the aligned sequences are marked with colored lines. The extent of the genes is marked in yellow. The probe used to detect the sequence in clinical validation of SSSD is marked in red.

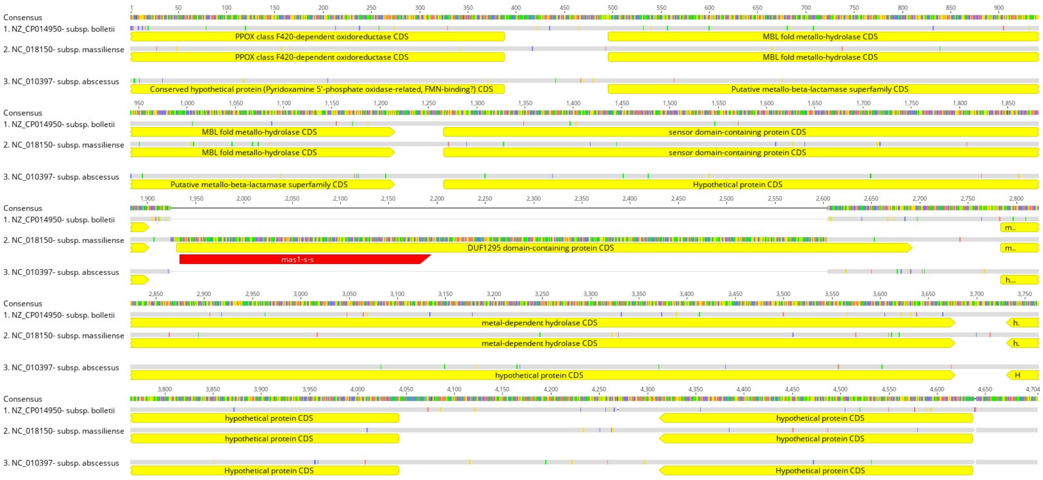

Fig. S6. The alignment between genomic regions surrounding (mas1-s-s) sequence in three reference genomes: NC_018150 representing *M. abscessus* subsp. *massiliense*, NZ_CP014950 representing *M. abscessus* subsp. *bolletii* and NC_010397 representing *M. abscessus* subsp. *abscessus*. Disagreements in the aligned sequences are marked with colored lines. The extent of the genes is marked in yellow. The probe used to detect the sequence in clinical validation of SSSD is marked in red.

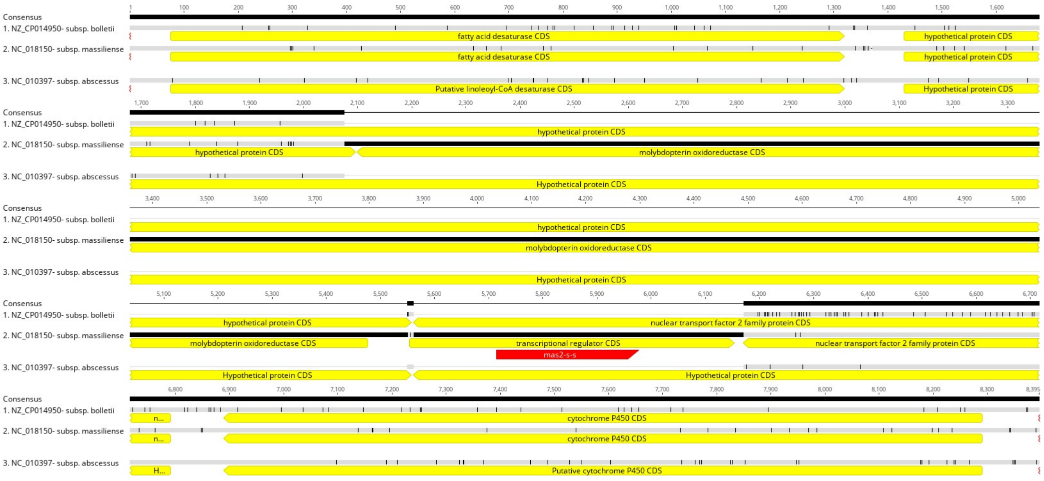

Fig. S7. The alignment between genomic regions surrounding (mas2-s-s) sequence in three reference genomes: NC_018150 representing *M. abscessus* subsp. *massilians*e, NZ_CP014950 representing *M. abscessus* subsp. *bolletii* and NC_010397 representing *M. abscessus* subsp. *abscessus*. Disagreements in the aligned sequences are marked with colored lines. The extent of the genes is marked in yellow. The probe used to detect the sequence in clinical validation of SSSD is marked in red.
